# Suppression of the gut microbiota–bile acid–FGF19 axis in patients with atrial fibrillation

**DOI:** 10.1111/cpr.13488

**Published:** 2023-04-26

**Authors:** Kun Zuo, Chen Fang, Yuanfeng Gao, Yuan Fu, Hongjiang Wang, Jing Li, Jiuchang Zhong, Xinchun Yang, Li Xu

**Affiliations:** ^1^ Heart Center & Beijing Key Laboratory of Hypertension, Beijing Chaoyang Hospital Capital Medical University Beijing China

## Abstract

This study aimed to investigate the role of the gut microbiota (GM)–bile acid (BA)–fibroblast growth factor (FGF) 19 axis in patients with atrial fibrillation (AF). Gut bacterial metabolisms of BAs were determined in an AF metagenomic dataset. The composition of faecal BAs pools was characterized by targeted metabolomics in an independent AF cross‐sectional cohort. Circulating levels of FGF19 were measured by ELISA. In vitro cell experiments were conducted to validate the regulatory role of FGF19 in atrial cardiomyocytes stimulated with palmitic acid. First, metagenomic profiling revealed that gut microbial biotransformation from primary to secondary BAs was dysregulated in AF patients. Second, the proportion of secondary BAs decreased in the faeces of patients with AF. Also, eight BAs were identified as AF‐associated BAs, including seven AF‐enriched BAs (ursodeoxycholic acid, chenodeoxycholic acid, etc.), and AF‐decreased dehydrolithocholic acid. Third, reduced levels of circulating FGF19 were observed in patients with AF. Subsequently, FGF19 was found to protect against palmitic acid‐induced lipid accumulation and dysregulated signalling in atrial cardiomyocytes, including attenuated phosphorylation of YAP and Ca^2+^/calmodulin‐dependent protein kinases II and secretion of interleukin‐1β, mediated via peroxisome proliferator‐activated receptor α. Our data found decreased levels of secondary BAs and circulating FGF19, resulting in the impaired protective function of FGF19 against lipid accumulation in atrial cardiomyocytes.

## Graphical abstract

In the current study, the BA synthesis‐related microbial signatures based on metagenome, the composition of the intestinal BAs pools based on metabolome, and the circulating FGF 19 level via ELISA were detected to characterize the profile of GM–BAs–FGF19 axis in patients with AF. Subsequently, the protective effect of FGF19 on palmitic acid‐stimulated HL‐1 cells in vitro was explored by evaluating the levels of lipid droplet accumulation, phosphorylated Yes‐associated protein (YAP) and Ca^2+^/calmodulin‐dependent protein kinases II (CaMKII) and secretion of interleukin‐1 β (IL‐1β) was revealed.

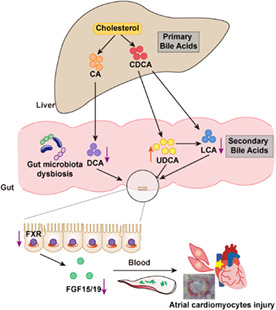

## 1 INTRODUCTION

With the increase in life expectancy in modern humans, atrial fibrillation (AF) has increasingly become a public health problem, intertwined with common concomitant cardiovascular risk factors, and recognized as cardiometabolic disease.^1^ However, the mechanism underlying the association between metabolism and the onset of AF needs to be fully understood.

**FIGURE 1:**
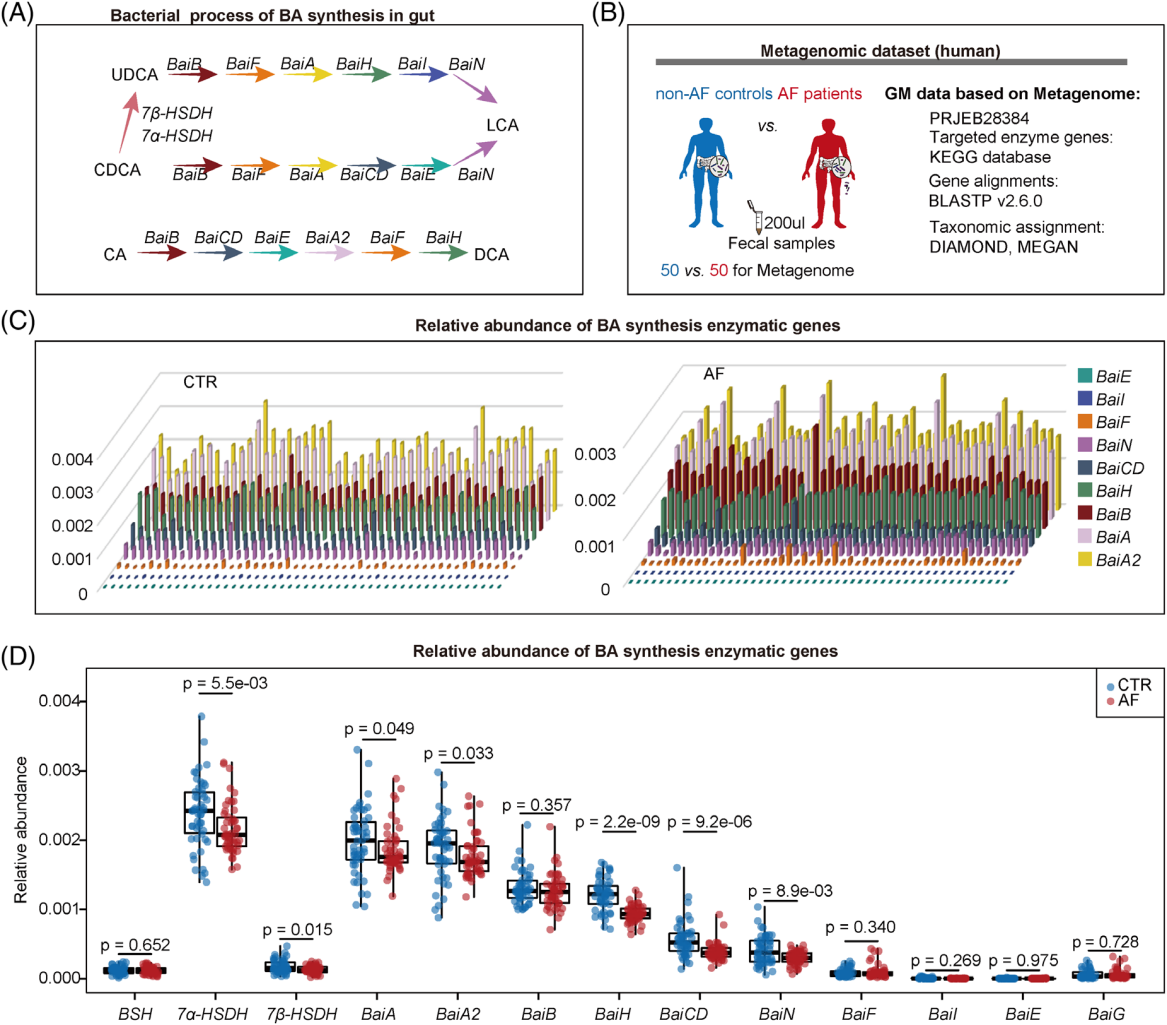
Altered bacterial enzymatic genes about BAs metabolism in the gut of patients with AF. (A) A simplified schematic shows the process of CDCA to UDCA, UDCA to LCA, CDCA to LCA and the dehydroxylation of CA to DCA. (B) Experimental design. The bar plot (C) and box plots (D) show the relative abundance levels of BAs‐related synthetic enzymatic genes.

The gut microbiota (GM) is an ecosystem containing billions of microorganisms that produce bioactive metabolites as potential regulators of AF pathogenesis.^2^, ^3^, ^4^ Preclinical and observational cohort studies have implicated an imbalanced GM in contributing to the development of AF. Among the recognized GM‐derived metabolites, BAs, which play roles in the modulation of host metabolism, inflammation and cardiovascular health,^5^ deserve special attention. Primary BAs, mainly cholic acid (CA) and chenodeoxycholic acid (CDCA), are synthesized from cholesterol in human hepatocytes and then biotransformed into secondary BAs by bacteria in the gastrointestinal tract^6^ (Figure 1A). For example, the epimerization reaction of CDCA to ursodeoxycholic acid (UDCA), catalysed by the enzymes 7α‐hydroxysteroid dehydrogenase (HSDH) and 7β‐HSDH, and the *bai* operon composed of nine enzyme genes mediated the conversion of CDCA and UDCA into lithocholic acid (LCA) and deoxycholic acid (DCA) from CA.

The regulation of BAs synthesis and transport was mediated by the nuclear farnesoid X receptor (FXR).^7^ Specific BAs bind to ileal FXR and regulate the expression of FGF19, which travels through the portal bloodstream and acts as a systemic metabolic messenger, such as the regulation of glucose and lipid metabolism.^8^, ^9^, ^10^ In addition, therapies targeting atrial lipid accumulation may potentially be beneficial for the treatment of AF.^11^, ^12^ Accumulation of lipids in the left atrial myocardium has been noted in patients with AF compared to patients with sinus rhythm.^13^ An increase in palmitic acid uptake was demonstrated in irregularly rhythmic myocytes compared to control or regular cardiomyocytes, which contributed to the activation of the atrial proapoptotic pathway.^13^ A prospective human cohort study suggests that higher plasma palmitic acid levels are associated with an increased risk of AF.^14^ Therefore, circulating FGF19 may play a role in linking the GM, BA pools and host metabolic homeostasis. Although the damage caused by GM‐derived BAs has been uncovered in several diseases, evidence for the role of a GM–BA–FGF19 axis in AF progression is still lacking, hampering the progress of future GM intervention strategies to target cardiac arrhythmias.

In the present study, microbial signatures characterized by metagenome‐based BA synthesis, the composition of metabolome‐based intestinal BAs pools, and circulating FGF 19 levels measured by ELISA were conducted to signify the profile of GM–BAs–FGF19 axis in patients with AF. Subsequently, the protective effect of FGF19 on palmitic acid‐stimulated HL‐1 cells in vitro was examined by evaluating the accumulation of lipid droplets, phosphorylation of Yes‐associated protein (YAP) and Ca^2+^/calmodulin‐dependent protein kinases II (CaMKII), and secretion of interleukin‐1 β (IL‐1β).

## 2 MATERIALS AND METHODS

### 2.1 Metagenomic cohort and enzymatic gene analyses

The metagenomic sequencing data of 50 patients with nonvalvular AF and 50 controls from northern China were analysed from a previous trial by our team.^4^ The exclusion criteria and the matched baseline characteristics of the 100 individuals have been deposited in the primary data.^4^ The bioinformatic assessment encompassing library construction, prediction of genes, taxonomic annotation and abundance determination was carried out as described in a previous report.^4^

The protein sequences of *BSH*, *7α‐HSDH*, *7β‐HSDH*, *BaiB*, *BaiF*, *BaiA*, *BaiH*, *BaiI*, *BaiN*, *BaiCD*, *BaiE*, *BaiA2* and *BaiG* were downloaded from http://www.ncbi.nlm. nih.gov/. The relative abundance levels of enzymatic genes and harbouring genera were obtained by aligning the nonredundant gene library to the sequences with BLASTP v2.6.0.^15^ First, a reference database was applied based on protein sequences of targeted enzymatic genes downloaded from the Kyoto Encyclopedia of Genes and Genomes (KEGG) database. Second, the enzymatic genes were identified by aligning nonredundant genes to the reference database using blastp. Then, the relative abundance levels of enzymatic genes were determined by summing the abundance of nonredundant genes annotated to the same enzyme. Finally, the taxonomic classification of enzymatic genes was executed according to the taxonomic annotation of related genes assessed from the previous analysis as follows. The genes were aligned to the integrated nr database to assess the taxonomic assignment using DIAMOND.^16^ The significant matches for each gene, defined by e‐values ≤10 × e‐value of the top hit, were determined and the retained matches were used to distinguish between taxonomic groups. The taxonomical level of each gene was determined using the lowest common ancestor‐based algorithm implemented with MEGAN (MEtaGenome Analyser).^17^ The abundance disparities of enzymatic genes and harbouring genera were assessed using the Wilcoxon rank‐sum test. A *p*‐value of <0.05 indicated a statistically significant difference.

### 2.2 UPLC‐MS/MS‐based faecal BAs quantification

BAs profiling and quantitation were performed using published methods with modifications. The faecal BAs were assayed using the ultra‐performance liquid chromatography–tandem mass spectrometry (UPLC‐MS/MS) system (ACQUITY UPLCXevo TQ‐S, Waters Corp., Milford, MA). The solvent and equipment preparation were as described previously. A total of 24 types of BAs were targeted for detection. BAs standards were bought from Steraloids, Inc. (Newport, RI) and TRC Chemicals (ON, Canada). Specific BAs chromatograms, as well as qualitative and quantitative ion mass and isotope internal standard parameters, were detailed in a previous study.^18^ The quality control samples were prepared with the test samples and injected at every 14 test samples throughout the process.

### 2.3 Cell culture

Mouse atrial cardiomyocytes (HL‐1 cells) and human intestinal epithelial cells (Caco‐2 cells) were purchased from BNCC (Henan, China) and cultured in Dulbecco's modified Eagle's medium containing 10% fetal bovine serum and 1% penicillin/streptomycin at 37°C with 5% CO_2_. Mouse HL‐1 cells treated with or without palmitic acid (PA; 200 μM; Sigma–Aldrich, St. Louis, MO), FGF19 (100 ng/mL; Proteintech) and PPARα inhibitor (10 μM) (GW6471, Selleck Chemicals) for 24 h. Human Caco‐2 cells were stimulated with or without LCA (100 μM) (MedchemExpress) and UDCA (100 μM; MedchemExpress) for 24 h. The cellular lysates and human Caco‐2 cell culture supernatant were then collected for subsequent experiments.

### 2.4 Measurement of FGF19 concentrations

The blood samples were collected from the antecubital vein in the fasting state using a vacutainer tube containing ethylenediaminetetraacetic acid (EDTA). After centrifugation at 3000 rpm and 4°C for 10 min, the plasma was separated and stored at ‐ 80°C in a 1.5‐mL microcentrifuge tube until analysis. FGF19 levels in plasma and human Caco‐2 cell culture supernatant were determined using an ELISA kit (KE00243, Proteintech) following the manufacturer's protocols.

### 2.5 Oil red O staining

The mouse HL‐1 cells were seeded in 12‐well plates, and after treatment for 24 h, Oil red O staining was carried out to assess intracellular lipid accumulation following the manufacturer's protocol. The cells were washed twice with phosphate‐buffered saline (PBS), fixed with 4% neutral formaldehyde for 30 min, and washed twice with double‐distilled water. Followed by treatment with 60% isopropanol for 30 s, freshly diluted oil red O solution was added for 20 min at room temperature. The cells were rinsed with 60% isopropanol for 30 s, washed thrice with PBS, and counterstained with haematoxylin for 2 min. After washing with double distilled water and rinsing with buffer for 1 min, the cells were observed and captured using a microscope.

### 2.6 Western blot analysis

After treatment, HL‐1 cells and Caco‐2 cells were lysed with RIPA buffer containing protease and phosphatase inhibitors, and proteins were collected after centrifugation at 13,000 rpm for 15 min at 4°C. The protein concentrations were determined using a BCA Protein Quantification Kit. Then, the cellular proteins were separated by sodium dodecyl sulphate–polyacrylamide gel electrophoresis and transferred onto nitrocellulose membranes. After blocking with 5% non‐fat milk, the membranes were incubated overnight at 4°C with the specific primary antibodies against PPARα, p‐YAP, IL‐1β, p‐CaMKII, CaMKII, Bax, Bcl‐2, FGF19, β‐tubulin and glyceraldehyde‐3‐phosphate dehydrogenase (GAPDH). The antibodies were obtained from Cell Signalling Technology and Proteintech. GAPDH and β‐tubulin were used as endogenous controls. After incubating with secondary antibodies for 1 h, the immunoblots were detected using an Odyssey infrared imaging system (LI‐COR, Lincoln, NE) and analysed using ImageJ software.

### 2.7 Statistical analysis

Continuous variables were expressed as mean ± standard deviation (SD) or median (quartile). The Student *t*‐test or Mann–Whitney *U* test was used to measure the difference in normally or non‐normally distributed data, respectively. The categorical variables were shown as a number or a percentage and compared using the chi‐square test. Pearson correlation analysis was performed to evaluate the correlation between left atrial diameter and plasma FGF19 level. The multivariable logistic regression analysis was applied to explore the relevant factors of AF based on the variables selected by univariate logistic analysis (*p* < 0.100) and diabetes mellitus (DM). All statistical analyses were carried out using SPSS 25.0 (SPSS) or R software (version 2.15.3). *p* < 0.05 indicated a statistically significant difference. All experiments were repeated at least three times.

## 3 RESULTS

### 3.1 Gut microbial biotransformation of primary BAs into secondary BAs, was decreased in AF patients

To investigate whether gut microbial BA synthesis was altered in patients with AF, specific bacterial genes coding for enzymes involved in secondary BA synthesis were identified from the metagenomic data of our previously published AF cohort (Figure 1B). The results showed that the relative abundance of microbial enzymatic genes for UDCA, LCA and DCA synthesis disrupted in the gut of patients with AF (Figure 1C, D), including *7α‐HSDH* (*p* = 5.5e‐03) and *7β‐HSDH* (*p* = 0.020), as well as the *baiA* (*p* = 0.049), *baiA2* (*p* = 0.033), *baiH* (*p* = 2.2e‐09), *baiCD* (*p* = 9.2e‐06) and *baiN* (*p* = 8.9e‐03).

**FIGURE 2:**
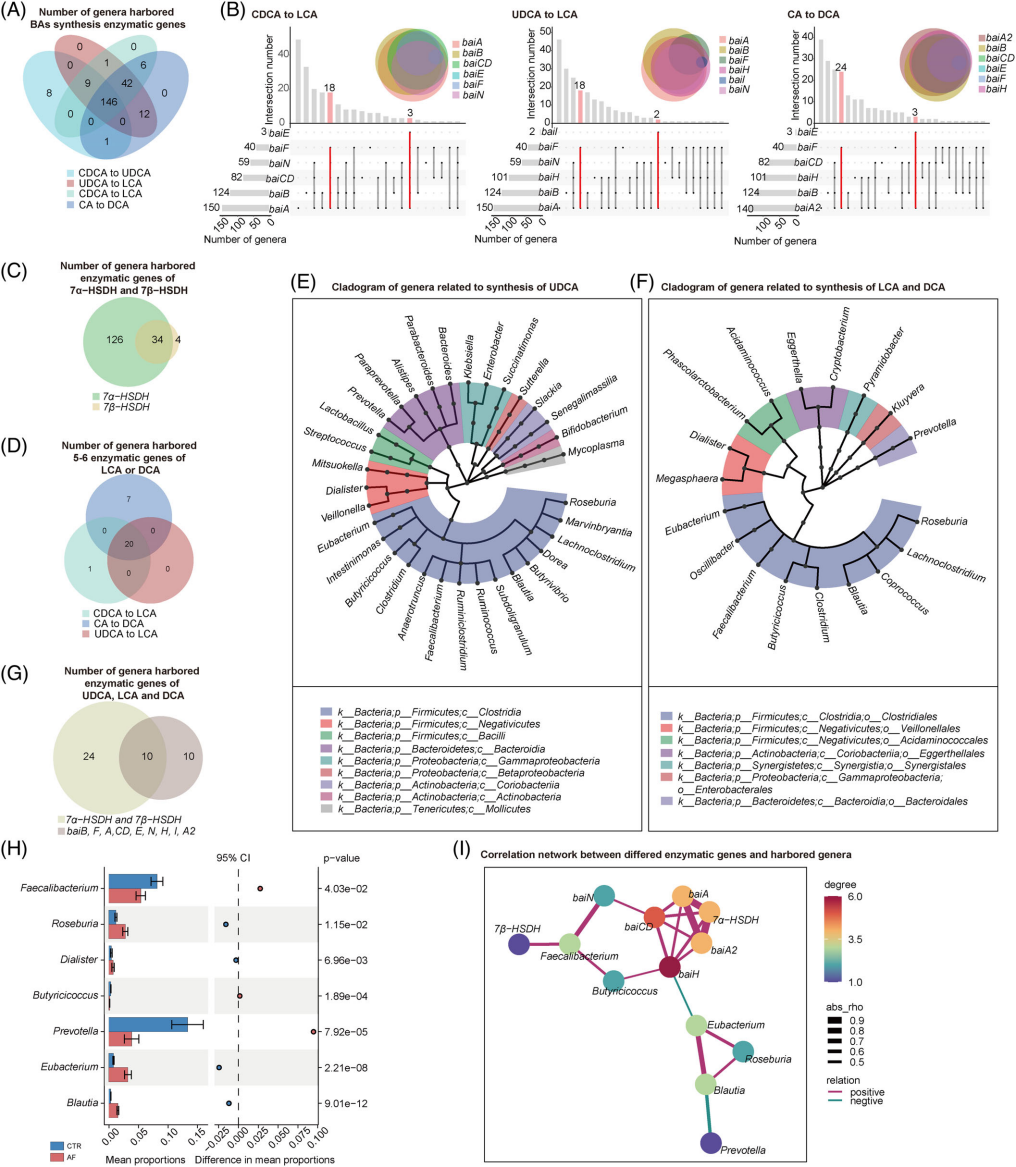
Gut microbes harbouring enzymatic genes related to BAs synthesis in AF. (A) Venn diagram showing the number of genera that harboured BAs enzymatic genes. (B) Upset Venn showing the distributions of genes in harboured genera. (C) Venn diagram showing the number of genera that harboured 7α‐HSDH and 7β‐HSDH genes. (D) Venn diagram showing the number of genera harbouring five to six enzymatic genes related to LCA or DCA synthesis. (E and F) Genera identified in panels C and D are shown in the phylogenetic tree. (G) Venn diagram showing the number of genera identified in panels C and D. (H) Stamp analysis showing the differential genera between controls and patients with AF. (I) Network analysis between differential BAs synthesis‐related genera and enzymatic genes.

Furthermore, the genes were aligned to the integrated nr database for evaluating taxonomic allocation to determine the signatures of intestinal bacterial organisms harbouring enzymatic genes related to BA metabolism in patients with AF. In the current metagenomic cohort, 164, 216 and 207 genera were defined as potential producers that harboured at least one gene related to UDCA, LCA and DCA synthesis, respectively (Figure 2A,B). In addition, 34 *7α‐HSDH‐* and *7β‐HSDH*‐harbouring genera (Figure 2C) and 20 *bai* operon (5–6 *bai* genes) genera (Figure 2D) were identified. Notably, many of these genera were annotated to the order of *Clostridiales* (Figure 2E,F). The relative abundance levels of 7 out of 10 genera that harboured genes related to UDCA, LCA and DCA synthesis (Figure 2G) were markedly differential in the gut of patients with AF (Figure 2H), including *Faecalibacterium* (*p* = 4.03e−02), *Roseburia* (*p* = 1.15e−02), *Dialister* (*p* = 6.96e−03), *Butyricicoccus* (*p* = 1.89e−04), *Prevotella* (*p* = 7.92e−05), *Eubacterium* (*p* = 2.21e−08) and *Blautia* (*p* = 9.01e−12).

Subsequently, the correlation network was used to explore the interaction between BA synthesis enzymatic genes and harboured genera in the AF cohort, and the complex linkages were shown (Figure 2I). For example, the abundance of *Faecalibacterium* remarkably decreased in patients with AF, which was positively correlated with AF‐deficient *baiN*. These data suggested the potential alteration of secondary BAs in patients with AF from the perspective of gut microbial BAs synthetic function.

### 3.2 Decreased secondary BAs in the feces of patients with AF

**FIGURE 3:**
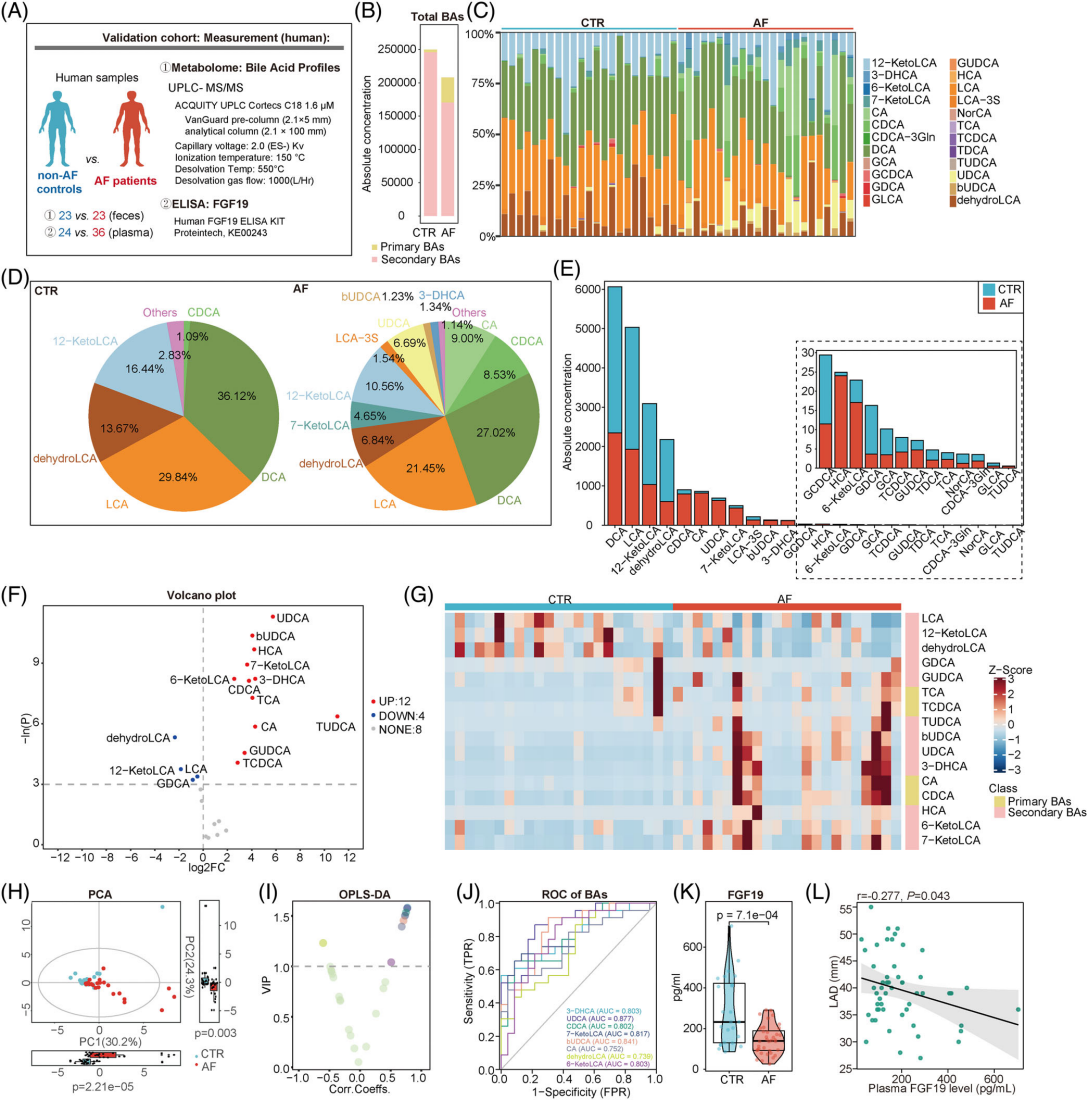
Dysbiotic bile acids profile and decreased FGF19 level in patients with AF. (A) Experimental design. Metabolomic profiling included the level of total bile acids (BAs) (B), the bar plot (C), pie plot (D) and histogram plot (E) of 24 BAs. The volcano (F) and heatmap (G) plots showed the different BAs between patients with AF and controls. (H) Principal component analysis models between controls and patients with AF. (I) Scatter plot about variable important in projection (VIP) based on the orthogonal partial least squares‐discriminant analysis (OPLS‐DA) models between controls and patients with AF. (J) Receiver‐operating characteristic (ROC) curve for the different BAs. (K) Plasma FGF19 level in patients with AF compared with non‐AF controls. (L) Pearson correlation analysis between FGF19 and left atrial diameter (LAD).

To validate whether the altered gut microbial BA synthetic function in AF patients led to an aberrant composition of faecal BAs, UPLC‐MS/MS‐based targeted metabolomics was performed to characterize the faecal BAs levels in an independent cohort consisting of 23 patients with AF and 23 non‐AF controls (Figure 3A). No significant difference was found between patients with AF and controls in terms of baseline characteristics, including age, gender, body mass index (BMI), hypertension, DM, serum cholesterol and liver or kidney function (Table 1).

**TABLE 1:** Baseline clinical characteristics of the participants with or without AF in the targeted metabolomics cohort.

	Control	AF	*P* value
Number	23	23	
Male (%)	11 (47.83%)	12 (52.17%)	1.000
BMI, kg/m^2^	26.65 ± 3.32	25.91 ± 3.58	0.544
HTN (%)	17 (73.91%)	15 (65.22%)	0.749
DM (%)	5 (21.74%)	1 (4.35%)	0.187
Smoking (%)	7 (30.43%)	6 (26.09%)	1.000
Drinking (%)	6 (26.09%)	5 (21.74%)	1.000
Age, year	62.48 ± 10.66	59.65 ± 12.45	0.413
TC, mmol/L	4.20 ± 0.88	4.20 ± 0.81	0.981
TG, mmol/L	1.39 ± 0.63	1.40 ± 0.96	0.964
AST, U/L	18.49 ± 7.29	19.37 ± 8.48	0.714
ALT, U/L	19.42 ± 11.95	18.35 ± 7.46	0.721
sCr, μmol/L	67.82 ± 12.62	66.56 ± 16.46	0.778

*Note*: Data are presented as mean ± SD, median (quartile), or number (%).

Abbreviations: ALT, Alanine aminotransferase; AST, aspartate aminotransferase; BMI, body mass index; DM, diabetes mellitus; HTN, hypertension; sCr, serum creatinine; TC, total cholesterol; TG, triglyceride.

Overall, 24 types of BAs were targeted for detection and quantification. Notably, the proportion of secondary BAs (*p* = 9.6e−05) decreased in patients with AF compared with controls (Figure 3B). Furthermore, the enrichment of 12 BAs was detected in the AF group, including UDCA (*p* = 1.2e−05), bUDCA (*p* = 3.1e−05), HCA (*p* = 6.2e−05), 7‐KetoLCA (*p* = 1.3e−04), 3‐DHCA (*p* = 2.7e−04), 6‐KetoLCA (*p* = 2.7e−04), CDCA (*p* = 3.0e−04), TCA (*p* = 6.9e−04), CA (*p* = 2.9e−03), GUDCA (*p* = 0.010), TCDCA (*p* = 0.020) and TUDCA (*p* = 1.7e−03). Meanwhile, four BAs levels were lower in AF than in control, including dehydroLCA (*p* = 4.9e−03), 12‐ketoLCA (*p* = 0.020), LCA (*p* = 0.030) and GDCA (*p* = 0.040; Figure 3C–G).

Then, the principal component analysis (PCA) was performed (Figure 3H), and the variable importance in projection (VIP) based on orthogonal partial least squares discrimination analysis (OPLS‐DA) was calculated to identify the BAs differing in AF. Based on the standard of VIP >1, eight BAs were identified as differed BAs in AF (Figure 3I). The receiver‐operating characteristic curve was conducted to compare the predictive value of BAs in AF. The results showed that the UDCA (area under curve [AUC] = 0.877, 95% CI: 0.780–0.975) had a significantly higher AUC compared with the 3‐DHCA (AUC = 0.803, 95% CI: 0.675–0.932), CDCA (AUC = 0.802, 95% CI: 0.674–0.929), 7‐KetoLCA (AUC = 0.817, 95% CI: 0.693–0.940), bUDCA (AUC = 0.841, 95% CI: 0.728–0.954), CA (AUC = 0.752, 95% CI: 0.610–0.895), dehydroLCA (AUC = 0.739, 95% CI: 0.596–0.882) or 6‐KetoLCA (AUC = 0.803, 95% CI: 0.673–0.933; Figure 3J). The optimal point for detecting AF of the UDCA was 35.195 nmol/g with a sensitivity of 87% and specificity of 78.3%. It followed that the altered faecal BA profiles in patients with AF were likely due to dysregulated gut microbial BAs synthetic function. However, the downstream regulatory effect of BAs on AF has remained unexplored.

### 3.3 Downregulation of circulating FGF19, a target of the secondary BAs, in AF patients

FGF19, an intestine‐derived hormone, was induced by the binding of BAs to ileal FXR and then released into the circulation, acting as an effector.^19^ To determine the downstream signalling of altered faecal BAs, circulating levels of FGF19 were measured by ELISA in 36 patients with AF and 24 controls. The specific baseline characteristics are presented in Table S1. No significant difference was found between the two groups in terms of demographic characteristics, hepatorenal function, and so forth. The results showed that the plasma FGF19 levels were significantly lower in patients with AF than in controls [232.779 (130.465–422.698) vs. 139.518 (92.555–189.668) pg/mL, respectively, *p* = 7.1e−04, Figure 3K]. During this period, a total of 11 non‐AF controls and 15 AF patients were evaluated for faecal BAs and plasma FGF19 levels, and a correlation analysis showed that plasma FGF19 levels were negative with faecal UDCA correlated (*r* = −0.174, *p* = 0.385) and positively correlated with faecal LCA (*r* = 0.120, *p* = 0.280). Due to the limited sample size, the correlation results were not statistically significant.

To determine the influence of significantly altered faecal BAs in AF patients (mainly LCA and UDCA) on the downstream production of the signalling molecule FGF19, human intestinal epithelial cells were cultured and stimulated with LCA or UDCA in vitro. The expression and secretion of FGF19 in human Caco‐2 cells were significantly induced by LCA treatment but inhibited by UDCA treatment (Figure S1).

Furthermore, a correlation analysis was performed in this cohort to evaluate whether a deficiency in circulating FGF19 was related to the development of AF. Left atrial diameter (LAD) was used as an indicator of AF development.^20^ We found that the circulating FGF19 levels significantly negatively correlated with the left atrial diameter (*r* = −0.277, *p* = 0.043, Figure 3L). Considering the correlation between FGF19 and DM, we included DM and variables selected by univariate logistic analysis (*p* < 0.100) in the multivariate logistic regression, indicating that the plasma FGF19 level was an independent protective factor for AF (OR = 0.992, 95% CI: 0.986–0.998, *p* = 0.011; Table 2). Thus, the GM–BA–FGF19 axis was suppressed in patients with AF.

**TABLE 2:** Association between variables and atrial fibrillation.

	Univariate analysis		Multivariate analysis	
	OR (95% CI)	*p*‐value	OR (95% CI)	*p*‐value
FGF19	0.990 (0.983–0.996)	0.001**	0.992 (0.986–0.998)	0.011*
LAD	1.180 (1.044–1.334)	0.008**		
LVEF	0.863 (0.761–0.978)	0.021*		
DM	1.250 (0.300–5.207)	0.759		

Abbreviations: LAD, left atrial diameter; LVEF, left ventricular ejection fraction; DM, diabetes mellitus.

* *p* < 0.05;

** *p* < 0.01.

### 3.4 FGF19 protected atrial cardiomyocytes from palmitic acid‐induced injury

**FIGURE 4:**
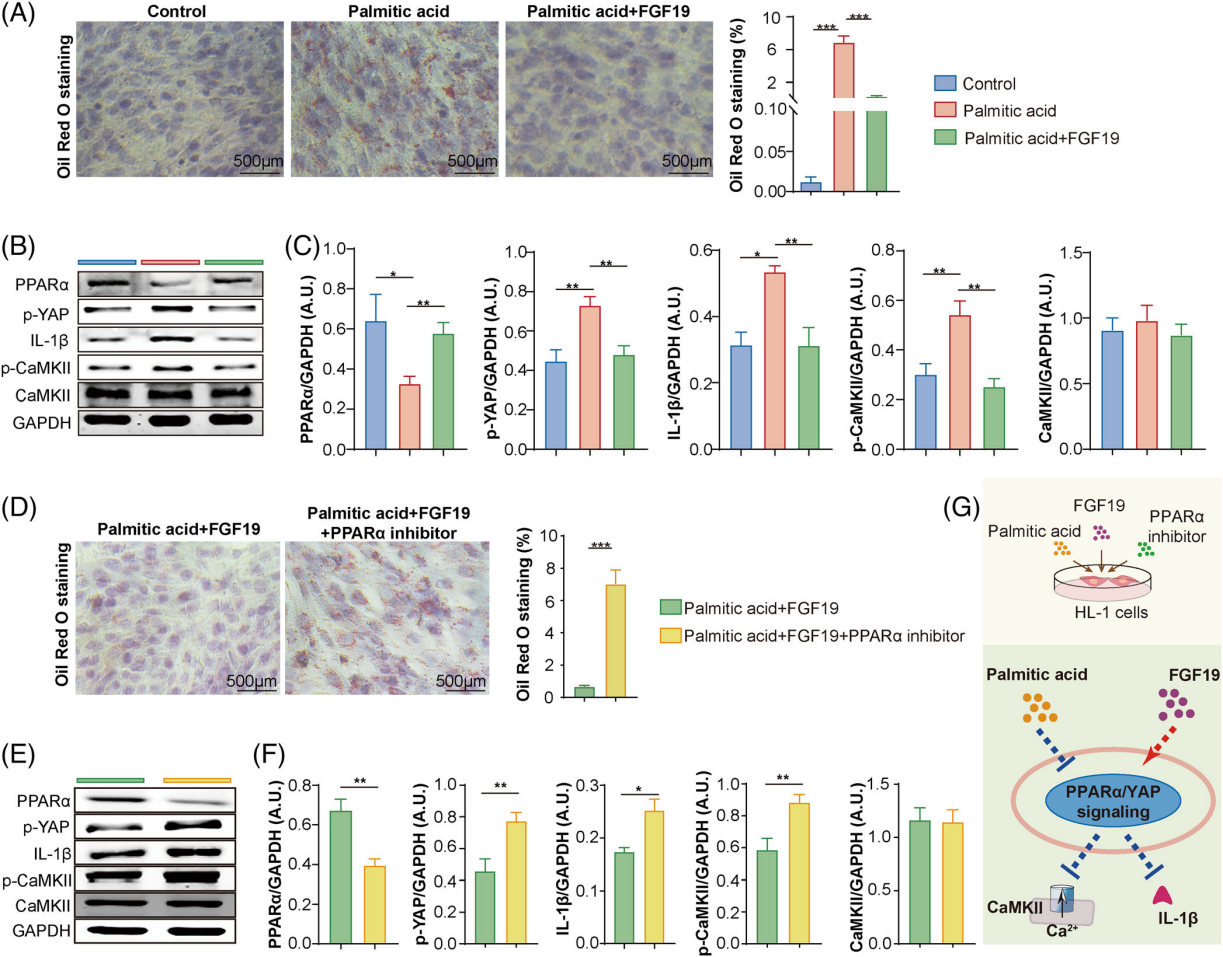
FGF19 alleviated atrial cardiomyocyte injury via PPARα signalling. (A) Representative Oil red O staining and statistical analysis for lipid droplets in cultured cells. *n* = 5–7. (B, C) Representative Western blot of PPARα, p‐YAP, IL‐1β, p‐CaMKII and CaMKII in HL‐1 cells treated with or without palmitic acid (200 μM) and FGF 19 (100 ng/mL). GAPDH as an endogenous control; *n* = 4–5. (D) Representative Oil red O staining and statistical analysis for lipid droplets in cultured cells. *n* = 4. (E, F) Representative Western blot of PPARα, p‐YAP, IL‐1β, p‐CaMKII and CaMKII in HL‐1 cells treated with or without palmitic acid (200 μM), FGF 19 (100 ng/mL) and PPARα inhibitor (10 μM). (G) Overview of the cell experimental design. GAPDH as an endogenous control; *n* = 4–6; *p < 0.05; **p < 0.01; ***p < 0.001.

We constructed a metabolic disorder model using HL‐1 mouse atrial cardiomyocytes stimulated with a long chain saturated fatty acid, PA, to investigate the correlation between FGF19 and the cardiometabolic phenotype of patients with AF.^21^, ^22^ After PA treatment, the lipid accumulation in mouse HL‐1 cells was increased, but this lipid accumulation could be attenuated by FGF19 treatment (Figure 4A).

Signalling pathways associated with atrial injuries were evaluated to elucidate the regulatory mechanisms of FGF19 in mouse atrial cardiomyocytes. Increased phosphorylation of YAP and the Ca^2+^/calmodulin‐dependent protein kinases II (CaMKII), as well as the expression of the pro‐inflammatory interleukin (IL)‐1β, were induced by PA, but alleviated by FGF19 treatment (Figure 4B,C).

A PPARα inhibitor was used to demonstrate whether the protective role of FGF19 against PA‐induced cell injury was mediated by PPARα signalling, which played a crucial role in lipid homeostasis and also regulated multiple physiological functions in cardiomyocytes, including inflammation and calcium handling.^23^

The results showed that the protective role of FGF19 in mouse HL‐1 cells was reversed by inhibition of PPARα activation, resulting in exacerbated lipid droplet accumulation, increased YAP and CaMKII phosphorylation, and IL‐1β secretion (Figure 4D–F). Meanwhile, increased Bax expression and decreased Bcl‐2 expression in palmitic acid‐stimulated mouse HL‐1 cells could be attenuated by FGF19 treatment (Figure S2A), which was further reversed by the intervention of the PPARα inhibitor (Figure S2B). These results suggested that FGF19 mitigated PA‐induced lipid metabolic disturbance and cellular injury via PPARα signalling in atrial cardiomyocytes.

## 4 DISCUSSION

Our study described the suppression of the GM–BA–FGF19 axis in patients with AF, encompassing a metagenomic cohort and an independent cohort to determine the levels of both faecal BAs and plasma FGF19. We increased the coverage of BA‐related enzymatic genes of gut bacteria and faecal BAs compared with a previous study on serum BAs in patients with AF.^24^, ^25^ Moreover, we demonstrated the decreased circulating FGF19 level in patients with AF and explored the protective role of FGF19‐PPARα on palmitic acid‐induced atrial cardiomyocyte injury. This study provided preliminary evidence for the concept of GM‐metabolized BAs, feedback on FGF19 secretion, and its regulatory effect on atrial cardiomyocytes. Our data might expand the recognition of BAs as bioactive molecules that mediated the effects of a dysregulated GM on AF progression and guide future GM intervention strategies that target heart arrhythmias.

Our multi‐cohort analyses revealed several biological phenomena. First, the gut microbial biotransformation of BAs, determined by enzymatic genes and harboured genera, was disordered in patients with AF. Our metagenomic investigation of the *bai* genes confirmed reads with high homology to gene sequences in the *bai* operon associated with DCA and LCA production.^26^ We further identified the exact taxa responsible for these decreased levels of secondary BAs in patients with AF compared with controls. However, strains with high homology to *Faecalibacterium* and *Butyricicoccus* might be determinant contributors, as they were shown to be reduced in patients with AF compared with controls. The overgrowth of *Roseburia*, *Eubacterium* and *Blautia* decreased with *BaiH*, which might be related to the decreased faecal DCA levels in AF. Taken together, we proposed that the disordered BAs‐related bacteria resulted in decreased levels of secondary BAs in the stool of patients with AF, which in turn exerted further biological effects.

Second, the composition of BAs was unbalanced in the faeces of patients with AF, while the disturbed BAs–FXR pathway might influence the host homeostasis. The binding of BAs to ileal FXR induced the expression of FGF19.^27^ BAs differed in their activation of FXR (CDCA > DCA > LCA >> CA), whereas UDCA acted as antagonists of the FXR/FGF19 system.^28^, ^29^ Thus, the altered BAs pool detected in the current AF cohort, such as the decreased DCA and LCA and enriched CA and UDCA, might partially explain the decreased FGF19 level in AF. How the interaction between BAs and FGF19 affects AF progression needs investigation.

Third, the decreased circulating FGF19 in patients with AF might prompt metabolic remodelling and energy metabolism disequilibrium in the development of AF.^30^ FGF 15 in mice and its human orthologue FGF19 (together denoted as FGF15/19) are gut hormones that serve as transversal metabolic coordinators at the crossroads of the gut, liver and other organs.^31^ Thus, the dysregulation of FGF19 signalling might contribute to the pathogenesis of several diseases. For example, FGF19 exhibited improved mitochondrial efficiency, which might be associated with higher cardiac contractility in hearts of patients with diabetes.^32^ FGF19 alleviates hypoxia/reoxygenation‐induced apoptosis and oxidative stress in cardiomyocytes.^33^ Consistently, the relatively deficient FGF19 and its negative correlation with left atrial enlargement were detected in patients with AF. Thus, it is possible that FGF19 plays a protective role during AF progression. In vitro, FGF15/19 intervention attenuated PA‐induced metabolic disorders and lipid accumulation in mouse atrial cardiomyocytes coupled with increased inflammation and the phosphorylation of CaMKII and YAP, which was reversed by the PPARα inhibitor. PPARα is a ligand‐activated nuclear receptor that regulates lipid catabolism and energy homeostasis. It acts as a physiological master switch in the heart, steering cardiac energy metabolism in cardiomyocytes, thereby affecting pathological heart failure and diabetic cardiomyopathy.^34^ Inflammation and CaMKII activation have been recognized as critical mechanisms of AF development.^35^, ^36^ Thus, it is possible that the dysregulation of FGF19/PPARα signalling is involved in AF progression.

Finally, this multi‐cohort study characterized the profile of GM–BAs pool–FGF19 in patients with AF and provided a preliminarily protective role of FGF19 in AF. Further studies are required to establish the intervention strategy targeting GM composition, which provides a potential therapeutic target for preventing atrial cardiomyocyte injury. For example, modulation of the GM with oligofructose enriches taxa involved in 6α‐hydroxylated BAs production and leads to Takeda G protein‐coupled receptor 5‐glucagon‐like peptide‐1 receptor axis activation to improve body weight and metabolism under western‐style diet feeding in mice.^37^

In summary, the present study on the suppressed GM–BA–FGF19 axis in patients with AF provided valuable resources and biological insights to facilitate future bacterial engineering and prebiotics‐based intervention medicine and enhance our understanding of the crosstalk between GM and cardiometabolic health.

There were some limitations with this study, including that it was a cross‐sectional study with small sample size and this study lacked an assessment of the concentrations and direct effects of circulating BAs on the development of AF. Further large‐scale prospective cohort studies and comprehensive mechanistic studies are therefore required.

## FUNDING INFORMATION

This study was supported by the National Natural Science Foundation of China (No. 82100334, 81970271, 81670214, 31771021, 92168117, 81770253 and 91849111), the Golden‐seed training plan (CYJZ202107) and Beijing Natural Science Foundation (7222068). Beijing Hospitals Authority Youth Programme (QML20230316).

## CONFLICT OF INTEREST STATEMENT

The authors declare that there are no conflicts of interest.

## Supporting information


**FIGURE S1.** (A) Representative Western blot of FGF19 in Caco‐2 cells treated with or without LCA and UDCA. GAPDH as an endogenous control; *n* = 3. (B) The levels of culture supernatant FGF19 in human Caco‐2 cells treated with or without LCA and UDCA. *n* = 3; *p < 0.05; **p < 0.01.
**FIGURE S2.** (A, B) Representative Western blot of Bax and Bcl‐2 in HL‐1 cells treated with or without palmitic acid, FGF19, and PPARα inhibitor. β‐tubulin as an endogenous control; *n* = 3; *p < 0.05; **p < 0.01.
**TABLE S1.** Baseline clinical characteristics of the participants with or without AF.

## Data Availability

Raw data was available at European Nucleotide Archive via PRJEB28384; the datasets generated and analyzed in this study are available from the corresponding author upon reasonable request.
