# Supplementary material for: Suppression of the gut microbiota–bile acid–FGF19 axis in patients with atrial fibrillation
Source: Cell Prolif. 2023 Apr 26;56(11):e13488. doi: 10.1111/cpr.13488 (PMC10623955; doi:10.1111/cpr.13488)
Supplement: Supplementary file 1 — FIGURE S1. (A) Representative Western blot of FGF19 in Caco‐2 cells treated with or without LCA and UDCA. GAPDH as an endogenous control; n = 3. (B) The levels of culture supernatant FGF19 in human Caco‐2 cells treated with or without LCA and UDCA. n = 3; *p < 0.05; **p < 0.01. FIGURE S2. (A, B) Representative Western blot of Bax and Bcl‐2 in HL‐1 cells treated with or without palmitic acid, FGF19, and PPARα inhibitor. β‐tubulin as an endogenous control; n = 3; *p < 0.05; **p < 0.01. TABLE S1. Baseline clinical characteristics of the participants with or without AF. [file CPR-56-e13488-s001.docx]

**TABLE S1.** Baseline clinical characteristics of the participants with or without AF.

|  | Control | AF | *P* value |
| --- | --- | --- | --- |
| Number | 24 | 36 | / |
| Male (%) | 8 (33.33) | 18 (50.00) | 0.288 |
| BMI, kg/m^2^ | 25.00 ± 3.23 | 25.32 ± 3.79 | 0.764 |
| HTN (%) | 15 (62.50) | 18 (50.00) | 0.430 |
| DM (%) | 3 (12.50) | 7 (19.44) | 0.725 |
| Smoking (%) | 6 (25.00) | 6 (16.67) | 0.517 |
| Drinking (%) | 6 (25.00) | 7 (19.44) | 0.751 |
| Age, years | 61.88 ± 10.64 | 65.00 ± 10.37 | 0.266 |
| TC, mmol/L | 4.41 ± 1.13 | 4.13 ± 1.01 | 0.354 |
| TG, mmol/L | 1.50 ± 0.71 | 1.27 ± 0.45 | 0.194 |
| AST, U/L | 18.16 ± 6.02 | 20.61 ± 7.34 | 0.200 |
| ALT, U/L | 15.00 (10.50, 19.00) | 18.00 (14.00, 25.50) | 0.061 |
| sCr, μmol/L | 66.18 ± 16.68 | 69.64 ± 15.30 | 0.428 |
| FBG, mmol/L | 5.14 ± 1.40 | 4.90 ± 0.87 | 0.435 |
| HbA1c, % | 5.70 (5.55, 6.20) | 5.80 (5.70, 6.30) | 0.195 |

**Abbreviations:** Data are presented as mean ± SD, median (quartile) or number (%). ALT, alanine aminotransferase; AST, aspartate aminotransferase; BMI, body mass index; DM, diabetes mellitus; FBG, fasting blood glucose; HTN, hypertension; HbA1c, hemoglobin A1c; sCr, serum creatinine; TC, total cholesterol; TG, triglyceride.


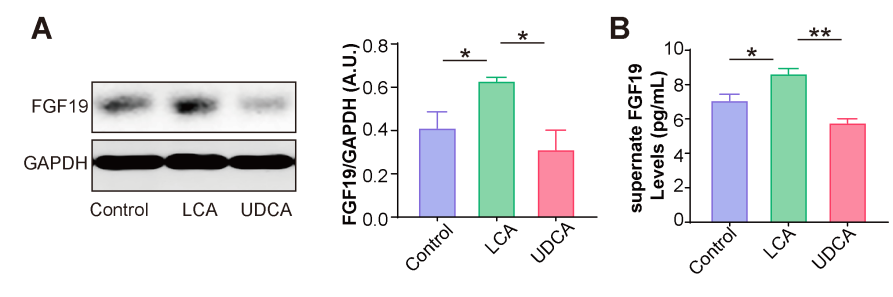


**FIGURE S1. (A)** Representative Western blot of FGF19 in human Caco-2 cells treated with or without LCA and UDCA. GAPDH as as an endogenous control; n=3. **(B)** The levels of culture supernatant FGF19 in human Caco-2 cells treated with or without LCA and UDCA. n=3; *, *P*<0.05; **, *P*<0.01.


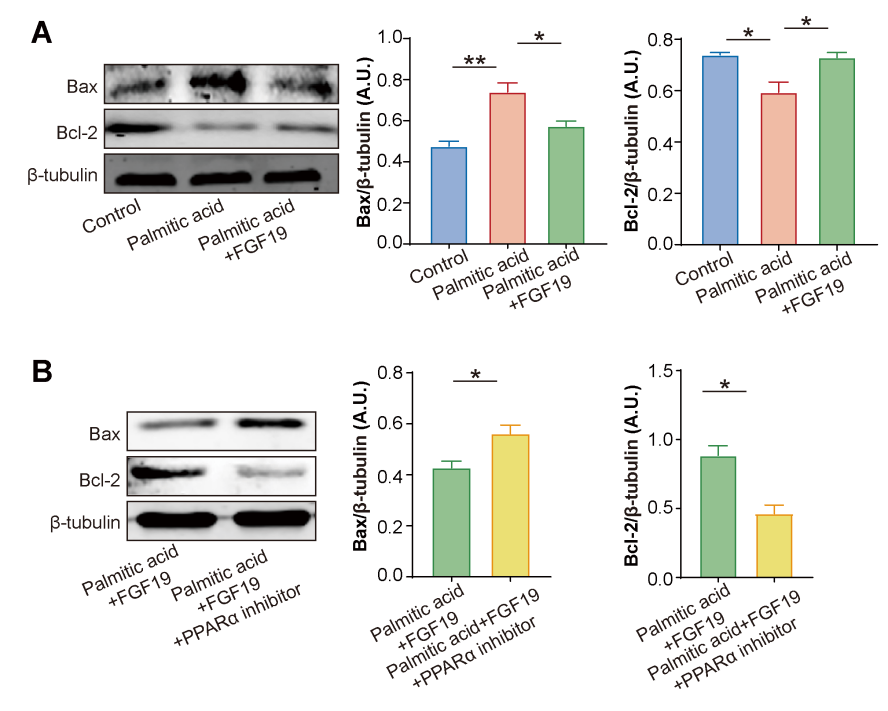


**FIGURE S2.** **(A-B)** Representative Western blot of Bax and Bcl-2 in mouse HL-1 cells treated with or without palmitic acid, FGF 19, and PPARα inhibitor. β-tubulin as as an endogenous control; n=3; *, *P*<0.05; **, *P*<0.01.
